# Laser-guided energetic discharges over large air gaps by electric-field enhanced plasma filaments

**DOI:** 10.1038/srep40063

**Published:** 2017-01-05

**Authors:** Francis Théberge, Jean-François Daigle, Jean-Claude Kieffer, François Vidal, Marc Châteauneuf

**Affiliations:** 1 Defence R&D Canada, Valcartier Centre, Québec, G3J 1X5, Canada; 2 INRS-EMT, Varennes, J3X 1S2, Canada

## Abstract

Recent works on plasma channels produced during the propagation of ultrashort and intense laser pulses in air demonstrated the guiding of electric discharges along the laser path. However, the short plasma lifetime limits the length of the laser-guided discharge. In this paper, the conductivity and lifetime of long plasma channels produced by ultrashort laser pulses is enhanced efficiently over many orders of magnitude by the electric field of a hybrid AC-DC high-voltage source. The AC electric pulse from a Tesla coil allowed to stimulate and maintain the highly conductive channel during few milliseconds in order to guide a subsequent 500 times more energetic discharge from a 30-kV DC source. This DC discharge was laser-guided over an air gap length of two metres, which is more than two orders of magnitude longer than the expected natural discharge length. Long plasma channel induced by laser pulses and stimulated by an external high-voltage source opens the way for wireless and efficient transportation of energetic current pulses over long air gaps and potentially for guiding lightning.

Electricity is now a domestically produced energy source that will transform our transportation sector. To address the growing need for an efficient power distribution, researchers were dreaming since two centuries to transmit electric energy over long distances in atmosphere. One of the most famous tentative was done one century ago by Nicolas Tesla with his alternative current (AC) high-voltage source^1^,^2^ named today to his name, i.e. the Tesla coil.

Experiments performed during the last decades confirmed that ultrashort laser pulses could produce plasma filaments in air that act like electrical wires^3^,^4^,^5^,^6^,^7^. These results on laser-guiding of pulsed direct current (DC) high-voltage demonstrated an increase of the discharge length relative to the natural breakdown gap by a factor of 1.3 to 1.5 only for long air gap^3^,^6^. On the other hand, recent results using pulsed Tesla coil sources showed more significant effect and the laser-guided discharge length was increased fourfold^8^,^9^. These works have mainly been motivated by triggering of lightning from storm clouds^10^ and to transport electric charges to a specific position. The initial laser-produced plasma channels used for these results are generated in air when the laser peak power exceeds a threshold named the critical power for self-focusing^11^. Such threshold is easily achieved with solid-state lasers and fibre lasers^12^ based on Chirped Pulse Amplification (CPA) techniques^13^. During their propagation in air, these ultrashort and intense laser pulses self-focus on themselves up to laser intensity sufficiently high to ionize the air. Starting from this zone, an automatic dynamic equilibrium occurs mainly between the laser self-focusing, plasma generation, plasma defocusing, laser diffraction, and air dispersion, which gives rise to thin and long plasma channels left behind the laser path^14^,^15^,^16^. Recently, deviations of arc discharges^17^ over a short distance were also demonstrated by using curved plasma filaments produced by beam-shaped ultrashort lasers.

The lifetime of long and high-density plasma channels is a critical parameter for electric arc control since it limits the maximum length that a laser can guide a discharge. The laser-produced plasma lifetime is very short, and almost independently of the initial plasma density, the free electron density will be reduced to below 10^11^ cm^−3^ in less than 1 *μ*s after the laser pulse in a cold plasma^18^. According to the traveling speed of laser-guided discharges^6^, which is around 100 km/s, a plasma lifetime of 1 *μ*s will allow to guide discharges up to few tens centimeters only. Recent works succeeded to increase the laser-produced plasma lifetime up to few microseconds by using an external electric field from a 50 kV DC high-voltage source^19^. In addition, other work recently reported an increase of the laser-guided discharge lifetime up to 130 *μ*s by inserting inductances on a 100 kV Marx generator^20^.

In this paper, we demonstrate the possibility to produce very conductive channels in air, to extend the plasma lifetime by three orders of magnitude (up to the millisecond scale) and laser-guide the discharge over an atmospheric gap 230 times longer than the natural discharge length of 0.88 cm for a 30-kV DC high-voltage source. These results are obtained by introducing a low energy pulsed electric field from a Tesla coil in parallel to the plasma filaments generated by ultrashort laser pulses. The Tesla coil electric field stimulated the plasma filament produced by the laser pulse and allowed the generation of a very conductive channel along the laser path, guiding the subsequent 30-kV DC discharge up to an atmospheric gap of 200 cm.

## Methods

The hybrid AC-DC high-voltage source (model 2MTC from ATTI^21^) used in this paper consists of the last generation of solid-state Tesla coil combined in series with a high load 30 kV capacitance allowing to store 500J of electric energy. The actual Tesla coil consists of two inductively resonantly coupled inductance-capacitance-circuits generating a 1J electric pulse (see Supplementary Fig. S1 for the main electrical components and data acquisition equipment). Ultrashort laser pulses of 0.15J from a portable CPA Ti:Sapphire system^22^ were focused using 4-m focal length lens to produce in air a bundle of conductive plasma filaments having a total length of 2.5 m. The conductive laser filaments were generated 1 cm above the electrodes. The filament bundle was positioned so that it started slightly before the high-voltage electrode and the highest plasma density filaments produced around the lens focus was positioned above the grounded electrode. The synchronization between the laser pulse and the AC waveform was optimized for the maximum laser-guided discharge length (>95%) over the 200-cm air gap. This was achieved by matching the laser-generation of plasma filaments with one peak of the Tesla coil electric field^8^,^9^, which accelerates the laser-produced free electrons along the plasma channel.

## Results

Before presenting the laser-guided discharges results, it is informative to quickly describe the phenomenology for a natural discharge, which is similar for both DC and AC high-voltage sources^23^. Similar to discharges from a lightning, the development of an electric discharge in air with a high-voltage source is initiated by the formation of a corona, followed by its self-constriction into multiple streamer formations, and finally, the most conductive streamer becomes the leader (see Supplementary Fig. S2 for more detailed descriptions)^24^. The leader, once initiated, progresses in the air gap at a speed of 10 km/s in successive sequences of formation of corona and streamers^6^. Finally, when the leaders coming from both electrodes have connected together, a short and intense current surge is transferred from the high-voltage source along the conductive leader channel and then the discharge occurs.

### Laser-guided AC discharges

**Figure 1:**
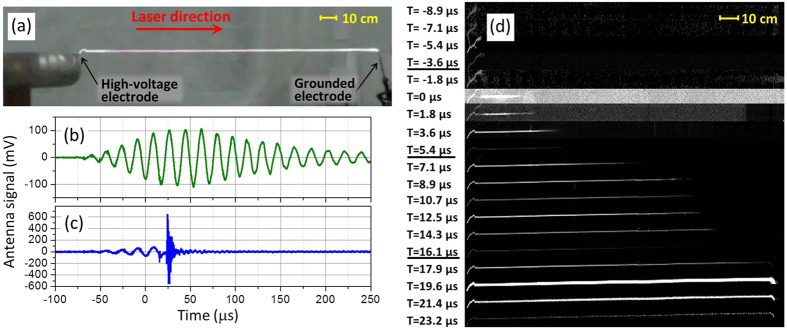
Laser-guided AC-discharge. (**a**) Time-integrated color image taken by a digital camera of the laser-guided 500 kV Tesla coil discharge over 200-cm air gap. (**b**) Temporal waveform of Tesla coil electric field detected by the antenna without firing the laser and (**c**) with the laser. (**d**) Successive video frames of laser-guided 500 kV Tesla coil discharge taken by a monochrome high-speed camera.

In the case of laser-guiding of the discharge, the plasma channel generated by the laser pulse bypasses the formation processes of coronas and streamers; consequently a leader will form and progress directly along the plasma filaments produced by the laser pulse. For these first results shown in Fig. 1, the DC high-voltage source connected to the high load capacitance (see Supplementary Fig. S1) was set to zero, and therefore these first results correspond to the phenomenology of laser-guided discharge from a purely AC high-voltage source (Tesla coil). In Fig. 1, the laser guiding was performed over an air gap of 200 cm, i.e. four times longer than the maximal length for non-guided discharge with the 500 kV Tesla coil used. Since the 200-cm air gap is much longer than the maximum non-guided breakdown gap, the electric field from the high-voltage source cannot initiate a corona on the grounded electrode, and therefore, the propagation of the leader is unidirectional and starts from the high-voltage source. It is very important to control this first step of laser-guiding of the AC discharge in order to succeed the guiding of the following energetic and longstanding DC discharge.

Figure 1a shows a time-integrated picture of the laser-guided AC discharge and Fig. 1d presents successive videos frames of the laser-guided leader progression taken by a monochrome high-speed camera. Figure 1b shows for reference the Tesla coil electric field detected by the antenna without laser. The full width at half maximum of the AC waveform pulse is 180 *μ*s and the average period is 18 *μ*s, corresponding to a Tesla coil resonant frequency of 55.6 kHz. Figure 1c presents the electric field amplitude emitted by the Tesla coil for the laser-guided discharge. The time zero corresponds to when the laser pulse passes above the Tesla coil and generates a bundle of plasma filaments between the electrodes. The radio-frequency noise starting around 20 *μ*s in Fig. 1c is produced by the abrupt AC discharge after the connection of the laser-guided leader between both electrodes. In Fig. 1d, the laser filament bundle was generated in the 200-cm air gap at the video frame labeled *T* = 0 *μ*s. The fluorescence from the laser-produced plasma was too weak to be detected by the high-speed camera, and the bright channel on the left-hand side of this video frame corresponds to the progression of the leader during the 1.8 *μ*s integration time per video frame. It is the scattering of the laser pulse from the wall rooms that produced the grey background on the video frame at *T* = 0 *μ*s. Before the laser pulse, as shown in Fig. 1b,c, the Tesla coil electric field already started to grow in amplitude. As a consequence, we can observe in video frames between *T* = −8.9 *μ*s up to *T* = −1.8 *μ*s in Fig. 1d the erratic initiation of streamers before the laser pulse arrives. Video frames with underlined label in Fig. 1d correspond to the time frames where the Tesla coil electric field goes to zero. Since the plasma and fluorescence lifetimes are very short^25^, the fluorescence ceases at these timings. However, because the electron-ion recombination transferred the electron energy to air molecules and induced significant temperature increase, a hydrodynamic expansion and a reduction of the air density occurred along the initial laser-induced plasma filaments. Some electrons trapped by O_2_ molecules create negatively charged molecules having a long lifetime^26^. Once the Tesla coil electric field increases again, these electrons can easily be detached from the parent molecules, and they are preferably accelerated along the low density channel created previously by the laser filaments stimulated by the Tesla coil electric field. The progression speed of the leader head along the laser path is 120 ± 20 km/s, which is ~12 times faster than for unguided discharges^6^. It took around 20 *μ*s (i.e. 1.1 Tesla coil cycles) to cross the 200-cm gap and once the leader gets close to the grounded rod, a non-guided streamer jumped the 1-cm vertical air gap from the ground rod up to the laser filament, finalizing the connection. Because of the low capacitance of the Tesla coil terminal and the low electric energy accumulated (1J), the following discharge stands only few microseconds (see video frames in Fig. 1d for 19.6 *μ*s < *T* < 23.2 *μ*s). However, this 1J discharge is energetic enough to heat more the channel initiated by the laser filaments, resulting in enhanced hydrodynamic expansion and a further reduction of the air density. The lower air density enhances the electrical conductivity because of less collision of electrons during their acceleration by an external electric field. Such increase of the air conductivity is crucial in order to realize the following step consisting of discharging the energetic 30-kV DC source over a gap 230 times longer than its natural breakdown length.

### Laser-guided AC-DC discharges

**Figure 2:**
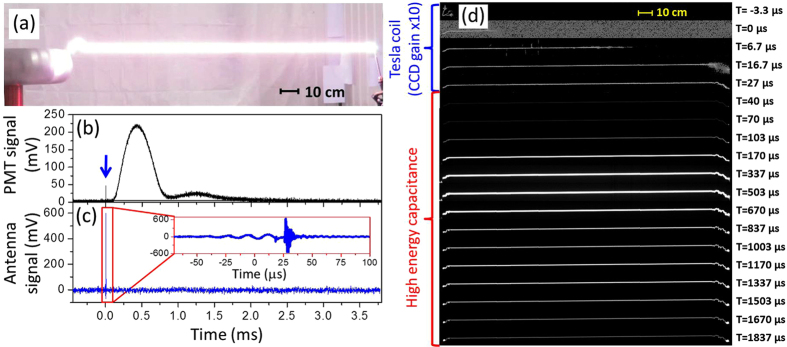
Laser-guided AC-DC discharge. (**a**) Time-integrated color image taken by a digital camera of the laser-guided AC-DC discharge over a 200-cm air gap. (**b**) Plasma fluorescence strength detected by a photomultiplier tube and (**c**) temporal waveform of the electric field detected by an antenna during the laser-guided AC-DC discharge. (**d**) Successive video frames of laser-guided AC-DC discharge taken by a monochrome high-speed camera.

Figure 2 presents the results for the same AC high-voltage parameters, but now a 30-kV DC voltage is applied on the high load capacitance connected at the base of the Tesla coil. In comparison with the results shown in Fig. 1, the plasma channel shown in Fig. 2a is much brighter and stands for few milliseconds instead of few microseconds, i.e. three orders of magnitude longer in time. Figure 2b presents the temporal evolution of the plasma fluorescence strength, which is nearly proportional to the square of the current flowing in the channel^27^. The short fluorescence peak around time zero in Fig. 2b and pointed out by the blue arrow above it corresponds to the fluorescence emitted by the leader and the discharge of the Tesla coil. It is important to note that the horizontal scale in Fig. 2b is in millisecond, and therefore, all events described previously in relation to Fig. 1 are concentrated in this narrow peak around time zero. Following the 1J Tesla coil discharge, the AC electric field vanishes after few tens of microseconds (see Fig. 2c and its inset), but a conductive and low air density channel remains along the laser path and this will drive the following 500J DC discharge from the high load capacitance. Because of the large inductance of the Tesla coil and the large capacitance connected to the DC high-voltage source, the resulting high impedance of this discharging circuit takes some time to build up the high-current making the fluorescence to peak 400 *μ*s after the laser pulse. This peak of fluorescence corresponds to a current around 80 A measured with a Hall effect amperemeter on the grounded electrode. It is interesting to note the absence of electromagnetic noise after the Tesla coil discharge in Fig. 2c and no radio-frequency noise was detected by the antenna during the 30-kV DC discharge. Such observation indicates that the air channel was very conductive and no sparking occurred during this DC discharge. Figure 2d presents successive video frames of the evolution of the laser-guided AC-DC discharge taken with a high-speed camera. In this configuration, we can observe the Tesla coil leader progression in three frames (0 *μ*s < *T* < 16.7 *μ*s) and the Tesla coil discharge along the laser path at frame *T* = 27 *μ*s. Shortly after the Tesla coil discharge, the plasma recombines and heats the air to a temperature of ~4800 K (determined from spectroscopic analysis) inducing a rapid hydrodynamic expansion of the heated air. Once the heated air expansion reduced enough the air density in the channel, a uniform glow discharge along the channel begins to drive the current from the 30-kV capacitance. The 30-kV discharge current flowing in the channel is uniform along the 200-cm air gap during the whole glow discharge (see video frames starting at *T* = 40 *μ*s down to the end). The bright spots observed on each electrode for video frames *T* > 837 *μ*s in Fig. 2d are the electrode incandescence due to the high current flowing at their extremities. It is important to note that without the AC high-voltage source activated, the 30-kV DC discharge would have been laser-guided over an air gap of 2–3 cm long only.

#### Spectroscopic analysis

**Figure 3:**
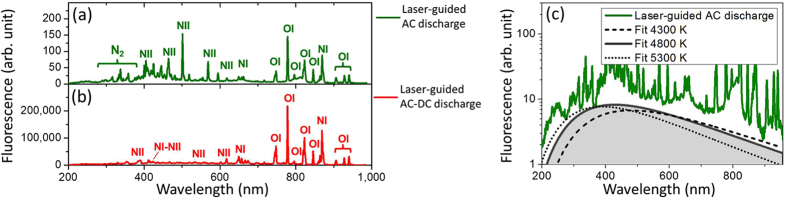
Typical fluorescence spectra of discharges. Fluorescence spectra from ultraviolet to near-infrared emitted by (**a**) the laser-guided AC discharge and (**b**) by the laser-guided AC-DC discharge. (**c**) Blackbody emission fits of the heated air after the laser-guided AC discharge.

Figure 3a,b present the fluorescence spectra from the plasma column at two steps during the hybrid AC-DC discharge. Figure 3a presents the fluorescence spectrum of the Tesla coil discharge, which is constituted mainly of singly ionized atomic oxygen (OI), singly (NI) and doubly (NII) ionized atomic nitrogen lines. Such spectrum indicates that more energetic electrons and fragmented air molecules were produced by the Tesla coil electric field during the laser-guided AC discharge. The fluorescence spectrum emitted during the DC glow discharge is presented in Fig. 3b, which is also constituted mainly of atomic oxygen and atomic nitrogen lines. However, the double ionization yield of nitrogen (NII) observed between 400 nm and 600 nm were lower relative to singly ionized fluorescence lines in the DC discharge (Fig. 3b) than for the AC discharge (Fig. 3a). This last observation indicates that lower excitation temperature was reached in the DC glow discharge as compared to the AC discharge. According to spectroscopic analysis, these measured fluorescence spectra correspond to excitation plasma energy around 2.6 eV (30 000 K) and 1.7 eV (20 000 K) for the spectra in Fig. 3a,b, respectively^27^,^28^. Such high excitation energies induce a drastic increase of the air temperature after the plasma recombination. According to the black-body emission fits obtained for the recorded thermal emission shown in Fig. 3c, the temperature increased to around 4800 K in the conductive channel after the laser-guided AC-discharge.

#### Discharge diameter and density

**Figure 4:**
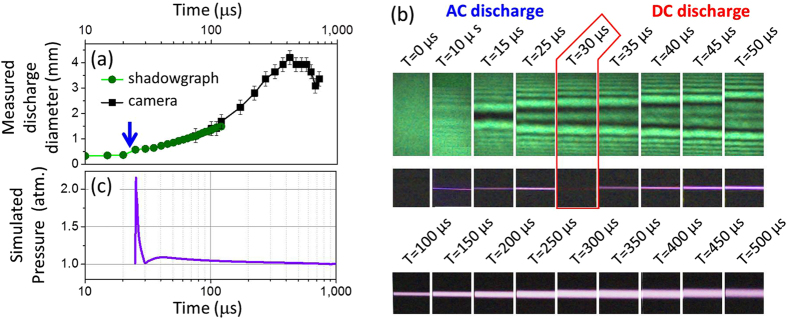
Laser-guided plasma channel diameter and pressure. (**a**) Laser-guided AC-DC discharge channel diameter as a function of time measured with a shadowgraph using a 532 nm green laser and with side-imaging high-speed color-camera. The blue arrow indicates the timing of the laser-guided AC-discharge. (**b**) Shadowgram patterns (first picture array) and real-color side-images (second and third arrays) of the laser-guided AC-DC discharge channel recorded with a high-speed color camera. (**c**) Simulated pressure inside the discharge channel as a function of time.

Figure 4a presents the measurement of the discharge diameter imaged directly by a real-color high-speed camera and by a shadowgraphy setup using a green laser beam at 532 nm that was crossing the discharge channel. These setups allowed to measure both the discharge channel diameter as a function of time and the plasma density during discharges^29^. The AC discharge occurred around time *T* = 25 *μ*s (high-speed camera integration time of 5 *μ*s) and the plasma density produced by the laser-guided AC discharge increased up to ~5 × 10^18^ electrons/cm^3^. After the AC discharge, this high density and energetic plasma recombines and the fluorescence ceases around *T* = 30 *μ*s as observed with the high-speed side-imaging in Fig. 4b. The electron pressure and gas pressure, which increased due to Joule heating, produced a sudden hydrodynamic expansion transverse to the channel axis^26^. Figure 4c shows a simulation of the pressure inside the channel using a homogeneous hydrodynamic model of the plasma channel^26^,^30^. The initial conditions for the DC discharge were determined by calculating the effect of a strong few microseconds current pulse (similar to the AC discharge observed in Fig. 1) producing a significant increase of the plasma conductivity (~1000 Siemens/m) due to the increase of the electron density and lowering of the gas density resulting from the channel expansion. Then by considering the measured impedance of the hybrid AC-DC high voltage source (see below) and the peak current measured during the DC discharge (80A), it was possible to simulate the channel pressure during the DC discharge starting shortly after the AC current peak. This simulation points out that the sudden pressure increase induced by the AC discharge was damped after a few microseconds by the shock wave generated in the ambient air. During the following DC discharge, the diameter of the glow discharge increased up to 4 mm at the peak current, but the pressure inside the DC discharge is only few percent higher than the surrounding atmospheric pressure (see Fig. 4c).

Shadowgrams and side-images in Fig. 4a,b show that few microseconds after the AC-discharge, the radius of the low density channel expands at a speed of 5 m/s (i.e. ~70 times slower that the speed of sound), confirming a quasi-equilibrium between the pressure inside the conductive channel and the surrounding ambient pressure. Considering this quasi-equilibrium and the air temperature of 4800 ± 500 K inside the channel after the laser-guided AC-discharge, these allow to estimate that the air density reduced by a factor of 16 ± 2 inside the channel with respect to the ambient air density.

#### Laser-guided breakdown voltage

The minimum DC voltage applied on the high load capacitance in order to observe the glow discharge shown in Figs 2 and 4 depends on the air gap length. Table 1 presents the DC voltage required for 50% probability of glow discharge at air gap ranging between 100 cm and 175 cm. Based on this measured DC voltage for 50% probability of glow discharge, the corresponding breakdown voltage is reduced to around 0.12 kV/cm, which is 230 times lower than the expected natural breakdown voltage at ambient air pressure and temperature. For all air gap lengths tested in Table 1, these measurements indicated that the laser-guided AC discharge generated a low density air channel that enhanced importantly the electrical conductivity because of less collision of electrons during their acceleration by the following DC electric field.

#### Laser-guided channel conductance

**Figure 5:**
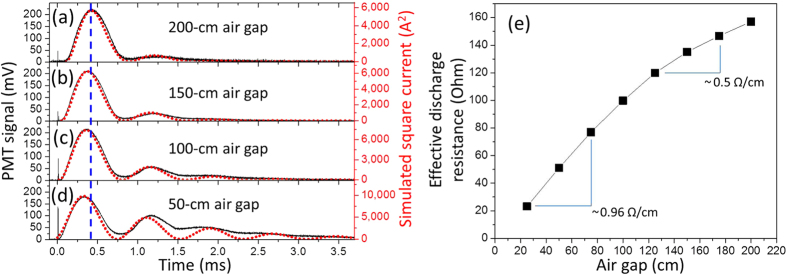
Temporal dependence of current flowing in plasma channels. (**a**–**d**) Measured temporal evolution of the AC-DC discharge fluorescence strength (black line) detected by the photomultiplier tube and calculated square current (red dotted line) for different air gap lengths. (**e**) Retrieved air resistance during the 30 kV DC discharge as a function of the air gap length.

The temporal evolution of the current flowing in the channel is governed by the conductivity (or resistance *R*_*air*_) of the air channel and by the inductance and the capacitance of the AC-DC high-voltage source. Figure 5a–d present the temporal evolution of the plasma fluorescence strength in the laser-guided AC-DC discharge for air gaps ranging from 200 cm down to 50 cm when a constant voltage of 30 kV was applied on the high-load capacitance. As noted earlier, the light luminosity of a discharge is nearly proportional to the square of the current flowing in the channel^27^. The oscillating structures observed for the fluorescence signals in Fig. 5a–d are due to the resonance of the inductance *L*_2_ and capacitances *C*_3_ of the high-voltage source (see Supplementary Fig. S1) and to the resistance of the air channel during the DC discharge. Such oscillating current corresponds to well-known underdamped resistive-capacitive-inductive (RCL) circuit, where the current flowing in the discharge channel is given by *I* = *I*_0_[exp(−*tR*_*air*_/2*L*_2_)sin(*tω*)]. In this expression, 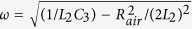, *t* is time and *R*_*air*_ is the air resistance in the low-density channel. Red dotted curves in Fig. 5a–d correspond to the simulated square current in the discharge considering a constant voltage of 30 kV on *C*_3_, constant values for the measured inductance *L*_2_ = 58 mH and capacitances *C*_3_ = 1.07 *μ*F. The value for the air channel resistance in the simulation was adjusted to obtain the best fit between the temporal evolution of the measured fluorescence (black curves) and the square of the simulated current (red dotted curves) in Fig. 5a–d. Recent works demonstrated that the resistance of air discharge channel remains mainly constant in time when using large-inductance high-voltage sources^31^. Therefore, as expected, time varying air resistances were tested for the simulation without significantly improving the fits. The values of the retrieved air channel resistances as a function of the air gap are shown in Fig. 5e. The effective air resistance slope in Fig. 5e ranges around 0.5 Ω/cm for long air gaps and increases to 0.95 Ω/cm for shorter air gaps. Such tendency is due to the use of fixed 30 kV on capacitance *C*_3_ while reducing the air gap length in Fig. 5a–d. Therefore, the breakdown can occur at a higher air density when reducing the gap length. As the channel expands after the AC-discharge, the subsequent DC-discharge can commence sooner in the more resistive (higher air density) channel for shorter gaps. This is the reason why the DC glow discharge in Fig. 5d was observed 100 *μ*s sooner than the discharge in Fig. 5a and that higher resistances were retrieved for shorter gaps. Once considering the effective resistance of the channel, its cross-section and its length, the conductivity of the channel after the AC-discharge is estimated to be around 1000 Siemens/m, which is similar to graphite conductivity^32^, but 19 orders of magnitude higher than non-ionized air at standard temperature and pressure^33^.

## Discussion

The success of laser-guiding an energetic DC discharge over an air gap 230 times longer than its natural breakdown length is due to a well-controlled sequence of laser-produced plasma filaments, followed by a plasma excitation from a Tesla coil discharge and the resulting hydrodynamic expansion of heated air allowing this longstanding DC glow discharge during few milliseconds. For the pulsed Tesla coil source, the use of ultrashort laser pulses to create a line of free charges in air through filamentation increases by 400% the maximum discharge distance from 50 cm to 200 cm. This is a larger effect than what has been measured with pulsed DC high-voltage sources^3^,^6^ where the laser-guided discharge length relative to the natural breakdown gap increased only around 30–50%. The maximum guided discharge length achieved in our trials was 200 cm. The length of the plasma filament bundle was 250 cm long and its plasma density was strong past the grounded electrode. Movement of the focusing lens position by decimeters did not impact the discharge length, so the limiting factor is not the length and plasma distribution of the laser filaments.

At ambient air pressure and temperature, the minimum electric field strength and longitudinal plasma density for the development of laser-guided leaders are around 4 kV/cm and 10^11^ electrons/cm, respectively^26^. Previous experiments in the literature showed that the maximum laser-guided discharge length is limited by temporal effects in the relation between the lifetime of the plasma filament, the pulsewidth of the high-voltage source and the propagation speed of the leader progressing along the laser path^4^. However, this works and others^19^ managed to show that the laser-produced plasma lifetime can be increased by using additional external electric fields. Therefore, these temporal effects are not restrictive in our experiment. The limiting factor in our experiments is the omni-directional emission of the high-voltage electric field from the Tesla coil which decreases as a function of the distance. As a consequence, when the electric field decreases below a threshold around 2.5 kV/cm to 4 kV/cm, the leader’s head cannot ionize more the air and its progression is ceased^34^,^35^. Such limit was reached in our experiment when the air gap was extended to 200 cm with the omni-directional emission of the Tesla coil electric field. However, the use of directional emission of electric-field like microwave beam or quadrupole phase-controlled Tesla coils could allow the stimulation of the laser-produced plasma channel over a longer distance.

Laser plasma filaments generated in air can reach a length of few hundreds of meters^36^. The results presented in this paper benefit for the lightning guiding as a ground-based directional electric-field source could be used to enhance the conductivity and lifetime of laser-produced plasma filaments in order to increase the probability of lightning triggering and guiding. In the case of laser triggering lightning from thunderclouds, the static electric field at the ground level^37^ reaches 5–15 kV/m. This natural electric field produced at ground level is similar to the DC breakdown voltage (~12 kV/m) achieved in this work and might be low enough to initiate glow discharge of lightning following the generation of conductive and low density air channels.

## Conclusions

The high excitation temperature of air after the laser-guided AC discharge induced a hydrodynamic expansion along the laser path and produced a conductive channel with an air density 16 times lower than for ambient conditions. Such highly conductive and low density channel reduced by a factor of 230 the breakdown voltage for the following DC discharge. High currents were flowing in these channels and the plasma channel diameter increased up to 4 mm during the DC glow discharge. The following stimulation of the laser-produced plasma channel was governed by electron acceleration from alternating electric field followed by plasma heating from Joule effect. The inverse bremsstrahlung process, by which electrons gain energy from the oscillating electric field, is proportional to the inverse of square frequency and makes the 55.6 kHz Tesla coil electric field 20 orders of magnitude more efficient for heating the plasma than other techniques using a second high-power laser beam having a wavelength in the visible or in the near-infrared. Finally, aside from increasing the probability of lightning triggering, these long lifetime plasma channels could be used also to increase by three-order of magnitude the emission time of previously demonstrated laser-guided plasma antenna^38^ and to increase by few orders of magnitude the microwave guiding distance^39^.

## Additional Information

**How to cite this article**: Théberge, F. *et al*. Laser-guided energetic discharges over large air gaps by electric-field enhanced plasma filaments. *Sci. Rep.* **7**, 40063; doi: 10.1038/srep40063 (2017).

**Publisher's note:** Springer Nature remains neutral with regard to jurisdictional claims in published maps and institutional affiliations.

## Supplementary Material

Supplementary Information

## Figures and Tables

**Table 1:** Measured air breakdown voltage for the DC glow discharge.

Atmospheric air gap	Measured DC Voltage for 50% probability of glow discharge	Breakdown electric field
(cm)	(kV)	(kV/cm)
100	12.5 ± 1	0.13 ± 0.01
125	15 ± 1	0.12 ± 0.01
150	17 ± 1	0.12 ± 0.01
175	23 ± 2	0.13 ± 0.01
