# Supplementary material for: Laser-guided energetic discharges over large air gaps by electric-field enhanced plasma filaments
Source: Sci Rep. 2017 Jan 5;7:40063. doi: 10.1038/srep40063 (PMC5214843; doi:10.1038/srep40063)
Supplement: Supplementary Information [file srep40063-s1.pdf]

Supplementary information:

Laser-guided energetic discharges over large air gaps by electric-field enhanced plasma filaments

Francis Théberge[1,*], Jean-François Daigle[1], Jean-Claude Kieffer[2], François Vidal[2], and Marc Châteauneuf[1]

[1]Defence R&D Canada, Valcartier Centre, Québec, G3J 1X5, Canada
[2]INRS-EMT, Varennes, J3X 1S2, Canada
[*]francis.theberge@drdc-rddc.gc.ca

Experimental setup

The hybrid AC-DC high-voltage source used in this paper consists of the last generation of solid-state Tesla coil combined in series with high load 30 kV capacitance. The primary circuit contains a large capacitance ($C_1 = 470$ nF) and a small inductance ($L_1 = 0.69$ mH), while the secondary circuit is a large inductance ($L_2 = 58$ mH) and a top load toroid with a small capacitance ($C_2 = 141$ pF). The primary circuit also includes a triggered high-voltage transistor to synchronize the primary circuit (jitter $<$100 ns) with the laser pulse. By inductive coupling, the primary circuit will generate an oscillating current in the secondary circuit (frequency of 55.6 kHz $= (1/2\pi)\sqrt{C_2 L_2}$) reaching a peak voltage of 500 kV on the top load toroid, corresponding to a maximal stored energy of 1 J. A 25-mm long metal rod (5-mm diameter) was fixed to the Tesla coil top load to provide a break-out point for the discharges and concentrate them to the point where the laser beam passed above. The high load capacitance C_3 in Fig. S1 can be charged up to 30 kV by a DC high-voltage power supply enabling to store up to 500 J of electric energy in this capacitance.

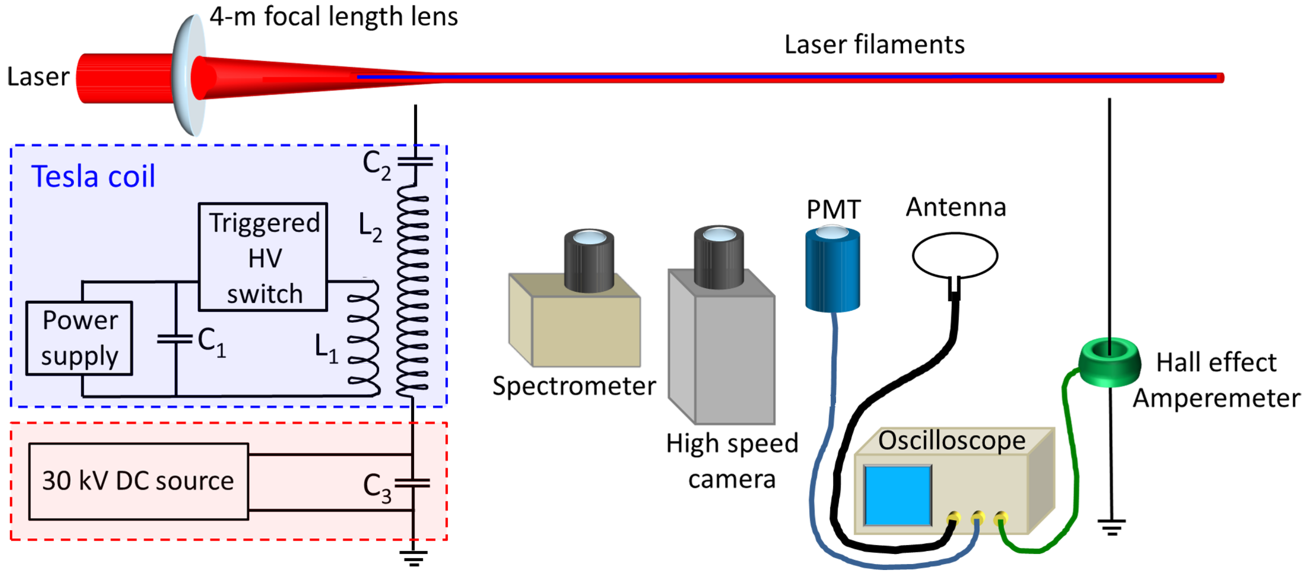

Figure S1. Experimental setup. The schematic of experimental setup for monitoring the laser-guiding of hybrid AC-DC high-voltage discharges. The AC high-voltage source is the Tesla coil in the blue-dashed box and the DC high-voltage source is connected to the high load capacitance C_3 in the red-dashed box.

Ultrashort laser pulses of 0.15 J from a portable Chirped Pulse Amplification Ti:Sapphire system[1] were negatively chirped to a pulsewidth of 1.02 ps (full width at $1/e^2$) and focused using 4 m focal length lens to produce plasma filament bundle of 2.5 m long in air. Laser filaments were generated 1 cm above electrodes and the filament bundle was positioned so that it started slightly before the Tesla coil electrode and the strongest part of the filament bundle produced around the lens focus was positioned above the grounded rod. During all measurements, laser filaments and the surrounding laser beam did not impact the electrodes.

Temporally resolved images of laser-guided discharges were captured using high-speed camera with time resolution down to few microseconds per video frame. In parallel, a photomultiplier tube (PMT) with a shorter time resolution of 1 ns was used to monitor the light emission from the discharge. Spectral distribution of the discharge emission was recorded using the spectrometer with an UV-grade collecting lens. Finally, an antenna was used to record the electric field emission and Hall effect Ampere meter was used to measure the current flowing in the grounded electrode.

Natural discharge phenomenology

Similar to discharges from a lightning, Figs. S2b-S2d present successive video frames taken by a high speed camera of a discharge induced by a Tesla coil without laser-guiding. The development of air discharge is initiated by the formation of a corona, followed by its self-constriction into multiple streamer formations (see streamers *A*, *B*, *C*, and *D* in Fig. S2b), and finally, the most conductive streamer becomes the leader (see streamer *D* in Fig. S2b becoming the leader path in Figs. S2c-S2d)[2]. The leader once initiated from the highly stressed electrode progresses in the gap in successive sequences of formation of corona and streamers. The leader head projects the electrical field from the electrode further into the gap, similar to introducing a conductive wire into the gap. In the case of moderate air gap with strong voltages as shown in Fig. S2, the projected electric field can be sufficiently high to initiate leaders on both electrodes, i.e. a negative and a positive leader. The progression mechanism of both positive and negative leaders is analog in air[3]. For the negative leader, it starts from the negative electrode and during its progression in the gap, the leader head is negatively charged, and vice-versa for the positive leader. Both require the generation of corona evolving into streamers before the progression of the leader, as seen in Figs. S2b-S2d for the corona halo between each leader. At each step of progression, new streamers are initiated from the head of the leader channel, and in front of these streamers, the projected electric field produces a corona halo in the air gap, which drive leaders progression. This corona will eventually constrict into new streamers (see streamers *E*, *F*, *G*, and *H* in Fig. S2c) which will make progress further the leader into the gap along an erratic path depending of initial impurities and their distributions in the air gap. This sequence of corona halo preceding the erratic progression of streamers can be well observed in Figs. S2b-S2d.

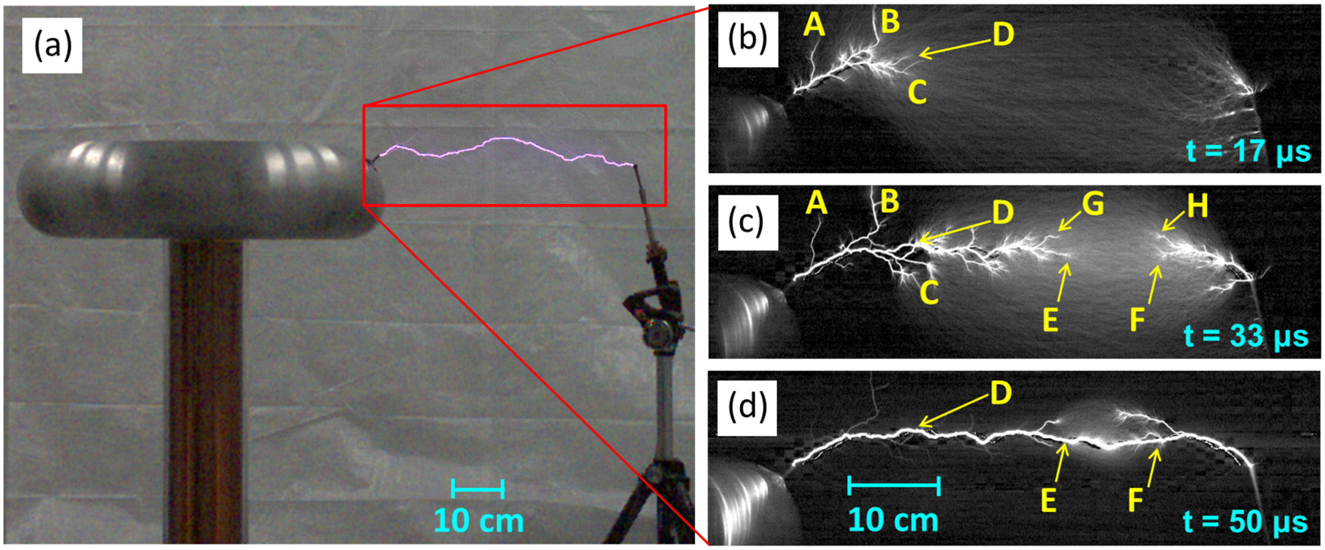

Figure S2. Discharge from a high-voltage source. (**a**) Color image taken by a digital camera of unguided 500 kV Tesla coil discharge over 50-cm air gap. (**b-d**) Successive video frames zoomed on the unguided Tesla coil discharge and taken by a monochrome high-speed camera. The gamma correction of video frames (**b**) to (**d**) was decreased to 0.5 to allow the observation of the corona halo.

Once the positive leader meets the negative leader in the air gap, the electrons at the negative leader head recombine with

the positive ions at the positive leader head and produce a brighter channel section at their intersection (see between leaders E and F in Fig. S2d). Finally, when leaders have connected completely the air gap, a short and intense current surge is transferred from the high-voltage source along the conductive leader channel and then the discharge occurs. If the air gap is further lengthened, the inducted electric charge on the grounded electrode is too weak to initiate a corona on this second electrode, and therefore for long air gap, there is only a single leader that progresses from the high-voltage electrode.

References

1. Châteauneuf, M. & Dubois, J. Canadian defense agency acquires new portable terawatt laser. *SPIE Newsroom* **10**, 0506 (2006).

2. Gallimberti, I., Bacchiega, G., Bondiou-Clergerie, A. & Lalande, P. Fundamental processes in long air gap discharges. *Comptes Rendus Physique* **3**, 1335–1359 (2002).

3. Raizer, Y. P. & Allen, J. E. *Gas discharge physics*, vol. 2 (Springer Berlin, 1997).
